# P-1709. Factors Associated with Anti-Methicillin-Resistant *Staphylococcus aureus* and Anti-*Pseudomonas aeruginosa* Therapy in Critically Ill Patients with Community-Acquired Pneumonia: A Single-Center Experience

**DOI:** 10.1093/ofid/ofae631.1875

**Published:** 2025-01-29

**Authors:** Nalea Trujillo, Calvin Diep, Ariadna Garcia, Marisa Holubar, David R Ha

**Affiliations:** Santa Clara Valley Medical Center, California; Stanford Health Care, Stanford, California; Stanford Health Care, Stanford, California; Stanford University School of Medicine, Stanford, CA; Stanford Health Care, Stanford, California

## Abstract

**Background:**

Current American Thoracic Society and Infectious Diseases Society of America community-acquired pneumonia (CAP) guidelines recommend empiric antibiotic therapy against methicillin-resistant *Staphylococcus aureus* (MRSA) or *Pseudomonas aeruginosa* (*P. aeruginosa*) in individuals with major risk factors: (1) MRSA or *P. aeruginosa* growth in respiratory cultures within the past year or (2) severe CAP with prior hospitalization and receipt of parenteral antibiotics within the preceding 90 days. The objective of this study was to describe factors associated with empiric antibiotic therapy against MRSA and *P. aeruginosa* in critically ill patients with CAP.

**Figure:**
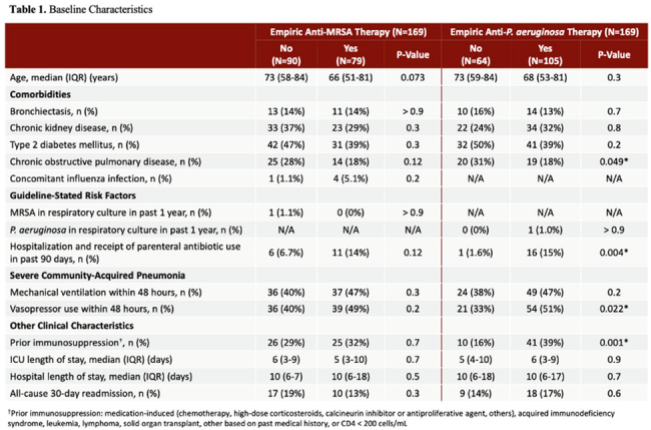

**Methods:**

This was a single-center retrospective study including adult patients admitted to the intensive care unit (ICU) with a diagnosis of pneumonia and who received systemic antibiotics solely for CAP from January 2022 to December 2023. The primary outcome was factors associated with empiric anti-MRSA and anti-*P. aeruginosa* therapy. Secondary outcomes included empiric antibiotic use relative to guideline-stated risk factors.

**Figure:**
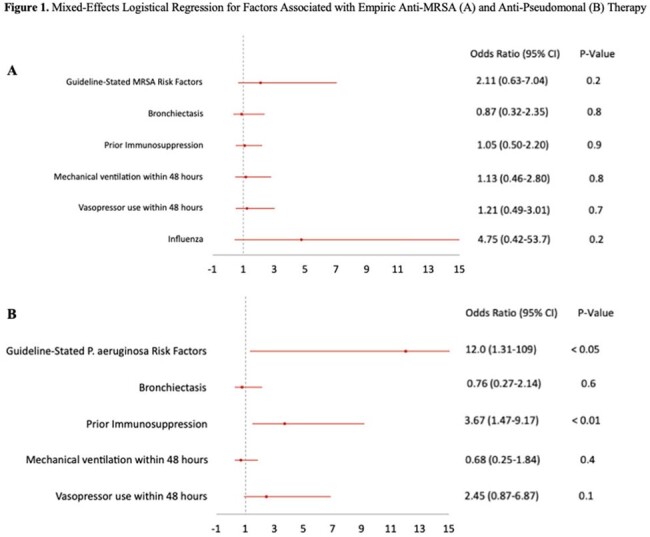

**Results:**

169 patients were included in the final analysis. No risk factors were associated with empiric anti-MRSA therapy. Multivariate regression analysis showed that the following were associated with empiric anti-*P. aeruginosa* therapy: presence of guideline-stated risk factors for *P. aeruginosa* (OR 12, 95% CI 1.31-109, p< 0.05) and prior immunosuppression (OR 3.67, 95% CI 1.47-9.17, p< 0.01). Prevalence of MRSA CAP was 1.8% and prevalence of *P. aeruginosa* CAP was 2.4%. Of the 79 patients who received empiric anti-MRSA therapy, 68 (86.1%) did not have guideline-stated risk factors. Of the 105 patients who received empiric anti-*P. aeruginosa* therapy, 88 (83.8%) did not have guideline-stated risk factors.

**Figure:**
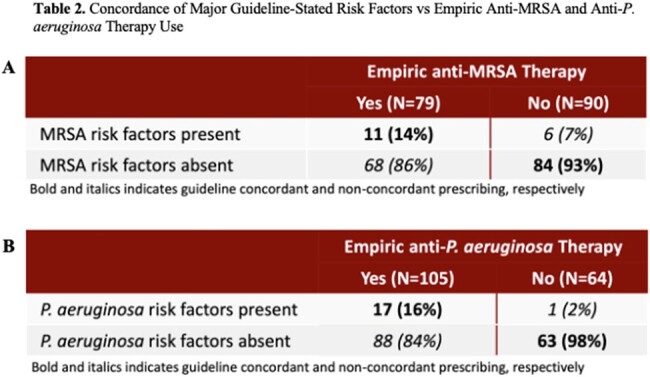

**Conclusion:**

Presence of guideline-stated *P. aeruginosa* risk factors as well as prior immunosuppression were associated with empiric anti-*P. aeruginosa* therapy and no factors were associated with empiric anti-MRSA therapy. Despite low incidence of MRSA and *P. aeruginosa* CAP, use of broad-spectrum antibiotics was frequent. Development of a protocol to guide empiric anti-MRSA and anti-*P. aeruginosa* therapy may help reduce unnecessary use of broad-spectrum antibiotics.

**Figure:**
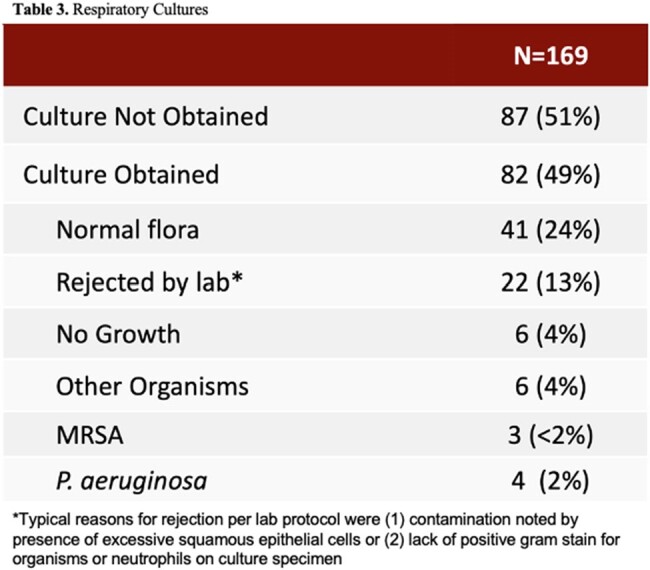

**Disclosures:**

**All Authors**: No reported disclosures

